# Case-mix adjustments for patient reported experience and outcome measures in primary care: an empirical approach to identify patient characteristics as case-mix adjusters based on a secondary analysis of an international survey among patients and their general practitioners in 34 countries

**DOI:** 10.1186/s41687-023-00667-8

**Published:** 2023-12-04

**Authors:** Peter P. Groenewegen, Peter Spreeuwenberg, Alastair H. Leyland, Dolf de Boer, Wienke Boerma

**Affiliations:** 1 Nivel – Netherlands Institute for Health Services Research, PO Box 1568, 3500BN Utrecht, The Netherlands; 2 MRC/CSO Social and Public Health Sciences Unit, Clarice Pears Building 90 Byres Road, Glasgow, G12 8TB UK

**Keywords:** Primary care, PREMs, PROMs, Case-mix, International comparison, Multilevel analysis

## Abstract

**Background:**

Case-mix adjustment of patient reported experiences (PREMs) and outcomes (PROMs) of care are meant to enable fair comparison between units (e.g. care providers or countries) and to show where improvement is possible. It is important to distinguish between fair comparison and improvement potential, as case-mix adjustment may mask improvement potential. Case-mix adjustment takes into account the effect of patient characteristics that are related to the PREMs and PROMs studied, but are outside the sphere of influence of the units being compared. We developed an approach to assess which patient characteristics would qualify as case-mix adjusters, using data from an international primary care study.

**Results:**

We used multilevel analysis, with patients nested in general practices nested in countries. Case-mix adjustment is indicated under the following conditions: there is a main effect of the potential case-mix adjuster on the PREM/PROM; this effect does not vary between units; and the distribution of the potential case-mix adjuster differs between units. Random slope models were used to assess whether the impact of a potential case-mix adjuster varied between units. To assess whether a slope variance is big enough to decide that case-mix adjustment is not indicated, we compared the variances in the categories of a potential case-mix adjuster. Significance of the slope variance is not enough, because small variances may be significantly different from zero when numbers are large. We therefore need an additional criterion to consider a slope variance as important. Borrowing from the idea of a minimum clinically important difference (MCID) we proposed a difference between the variances of 0.25*variance (equivalent to a medium effect size). We applied this approach to data from the QUALICOPC (Quality and costs of primary care in Europe) study.

**Conclusions:**

Our approach provides guidance to decide whether or not patient characteristics should be considered as case-mix adjusters. The criterion of a difference between variances of 0.25*variance works well for continuous PREMs and PROMs, but seems to be too strict for binary PREMs and PROMs. Without additional information, it is not possible to decide whether important slope variation is the result of either differences in performance between general practices or countries, or cultural differences.

**Supplementary Information:**

The online version contains supplementary material available at 10.1186/s41687-023-00667-8.

## Background

In this article we report on a secondary analysis, aimed at assessing case-mix controls for patient reported outcome measures (PROMs) and patient reported experience measures (PREMs) for primary care in international comparisons. Based on the literature, we will further develop the methodology and provide an application with real world data. New to this paper are our approach to distinguishing between fair comparison and improvement potential, and our consideration of case-mix controls in an international comparative context. We used data from the international QUALICOPC (Quality and costs of primary care in Europe) study, a cross-sectional, linked survey among approximately 70,000 patients and 7,000 general practitioners (GPs) in 34 countries, mainly situated in Europe [1].

Case-mix adjustment can be defined as a statistical procedure to account for differences in the mix of patients across units, in order to make fair comparisons of the relative performance of units (based on a definition of [2]). Units are comparable if, through statistical adjustment, they effectively treated a patient population with the same characteristics [3]. In general case-mix adjustment is used for two reasons: fair comparisons between health care providers, regions or countries, and identification of improvement opportunities by showing which aspects of care vary between providers or countries and thus are under the influence of the providers or countries. For both purposes, measures need to be made comparable between units within or between countries. The effect of variables related to the outcome, but beyond the influence of the units that are compared, should be adjusted for. On the other hand, the effect of those variables that are related to the outcome but lie within the influence of the relevant units should not be adjusted for, because there is apparently potential for improvement. Patient characteristics should be considered as a potential case-mix variable on the basis of statistical and theoretical reasoning. There should be an explanation for a relationship of the characteristic with the outcome studied. The effect of adjustments depends on the strength of the relationship and difference between units in the distribution of these variables. If a patient characteristic with a strong impact on the outcome is similarly distributed among units, the adjustment will have no impact. Not applying case-mix adjustment in case of a differing distribution may lead to the conclusion that some units perform better, while in reality the difference is only caused by the difference in distribution.

Case-mix adjusters concern the effects of variables that are beyond reasonable control of units. To what extent this is the case, can be debated. For example, when analysing PREMs, health and care capabilities of patients may influence their experiences with primary care. One may argue that these capabilities can be influenced by primary care providers. For example, care providers can adjust their communication with patents with low capabilities and in doing so lessen the difference in experience between patients with lower and higher capabilities. Others would perhaps argue that this is possible in principle, but that we cannot reasonably expect this of primary care providers, if only because of a lack of clear guidelines as to how to achieve this. In the first line of reasoning, health and care capabilities do not have to be controlled for, while in the second they should be considered case-mix adjusters to allow fair comparison. Which argument will be adopted depends on the purpose and question of the study, and how mechanisms behind the relationship between a patient characteristic and an outcome are understood. Consequently, often there will not be a clear dichotomy of variables that should or should not be used as case-mix adjusters; there might be a third category of variables that may be considered a case-mix adjuster or not, depending on the purpose of their use.

In this article we study these issues, using secondary analysis of primary care data. The context of this secondary analysis is the OECD PaRIS survey that aims to collect data on the PREMs and PROMs of patients with chronic conditions in primary care in OECD member states [4]. To the best of our knowledge an international analysis of case-mix adjustments for PREMs and PROMs in primary care has not been reported previously. The results will also be relevant for comparisons of patient outcomes in international studies in general.

### Selection of potential case-mix variables

Although, statistically, every variable can be a case-mix adjuster, there are good reasons to make an a priori selection. Obvious restrictions are the content of an existing database or the variables included in a survey. A selection of adjusters can be based on theoretical considerations, the conceptual framework of the study, and published literature on case-mix adjustments. Furthermore, case-mix adjusters should be applicable to several dimensions of PREMs and PROMs (and not just to one item) and have some face validity (e.g. based on previous research) [5]. A heuristic is to look for adjusters in the following categories: demographic, access to care, healthcare seeking behaviour, geographic location, clinical characteristics, and comorbidity [6]. This heuristic should be adapted to the context of a study (e.g. nature of the units and the services).

### Usual approach to adjustment

For a fair comparison between providers and countries the results of patient measures need to be corrected for relevant case-mix variables. Case-mix adjusters are usually selected with two criteria: the heterogeneity of the distribution over the units to be compared (differences in composition of the patient populations between units), and the strength of the relationship of an adjuster with the outcome [7]. Previous research has combined these criteria into an impact score [8–10].

### Varying relationship between potential adjuster and outcome

The existence of a relationship between patient characteristics and outcomes, and heterogeneity is not sufficient to qualify as case-mix adjusters. In addition to the strength of the relationship with the outcome the effect between units must differ, which means that some units have better outcomes than others [11, 12]. For example, it might be argued that PREMs should be corrected for age of the patients, if in the usual approach the age of patients is associated with their experiences of care and if the age distribution of the patients differs between units. However, the association between age and PREMs may also differ between units, suggesting that some units may ‘produce’ more favourable experiences at all ages, whereas others may do so for older patients only. The performance of units may not be the only explanation for a varying relationship. Also a differing response tendency and cultural differences can play a role, as we will explain in the discussion. It should be noted that quality differences that are within the realm of influence of units may be masked by adjusting for case-mix variables that have a varying relationship with the outcome. In terms of a multilevel statistical approach, this implies that there is significant random slope variation between units. This idea is not new [11, 13], but it is only rarely applied, for reasons unknown to us. Stratified analysis (e.g. mentioned by Iezzoni [2]; p.251; and by the National Quality Forum [14]) has been suggested. Also interaction terms between dummy variables for the units and potential case-mix adjusters have been used. [15] Stratified analysis and the use of interaction terms provide comparable information but in a less efficient analysis, particularly with large numbers of units.

### Case-mix adjustment in international research

We found no multiple country studies discussing the problem of case-mix adjustment for PREMs or PROMs. We identified a study comparing two countries in which methodological challenges were addressed but with little added value for our purpose. The authors mention the possibility that case-mix adjusters may differ between countries or health care systems [16].

### Research questions

This paper aims to develop an approach to guide decisions about potential case-mix adjusters in an international comparison, using random slope variation in multilevel analyses. We address the following research questions:

1. Which patient socio-demographic background and health status characteristics are associated to PREMs and PROMs in primary care; in statistical terms: do they have a main effect?
2. Which of these patient characteristics vary in their relation to PREMs and PROMs over primary care practices and countries and which of them do not; in statistical terms: does the slope of the relationship vary?
3. Is the variation in the relationship (the slope variance) between these patient characteristics and PREMs and PROMs too large to consider their use as case-mix adjusters?
4. Does the distribution of these potential case-mix adjusters differ between providers and/or countries?

## Methods

### Data

We used cross-sectional data collected in the QUALICOPC study between 2010 and 2012 [1]. For this study, primary care practices were sampled in 34 countries. As QUALICOPC was co-funded by the European Commission, the aim was to include the EU member states plus countries from the European Free Trade Association (Iceland, Norway, Switzerland) and pre-accession candidates that wanted to participate. Three non-European countries wanted to participate (with their own funding) to acquire comparative information from a large number of European countries. The 34 countries included the (then) EU 27—except for France, plus Iceland, FYR Macedonia, Norway, Switzerland, Turkey and Australia, Canada and New Zealand) [17]. Around 220 GPs per country participated, except for very small countries (Cyprus, Iceland, Luxembourg, and Malta) where this was around 75 GPs. For the UK, only GP practices in England were sampled. In Canada, Belgium, and Spain, larger samples were taken to represent different regions. In most countries, a random sample was invited to participate. Where no national sampling frame was available, alternatives were sought as close as possible to a random sample. Per practice, only one GP participated. The response among GPs was on average 30% and ranged from 6 to 90% between countries. The number of GPs invited to participate varied between 78 (Malta) and 5000 (Belgium) and the number of responders varied between 70 (Malta) and 553 (Canada). The response group mirrored the national GP populations in terms of age and sex [17].

Patient questionnaires were, in most countries, administered by fieldworkers in the waiting room right after the consultation with the participating GP. Ten consecutive patients were invited, nine of which filled out the patient experience questionnaire and one the patient values questionnaire (not used in this analysis). The average response rate of patients was 74% as reported by the fieldworkers, based on the numbers of patients that had to be invited before the target number of ten was reached, and ranged from 55 to 88% between countries. The absolute number of patients that filled out the patient experience questionnaire varied between 624 (Cyprus) and 5009 (Canada). [18]

### Measurement of PROMs and PREMs

To measure PROMs we used two questions: Self-rated health (wording of the question: How would you describe your own health in general? With answering options very good, good, fair, poor), and enablement (wording of the item: After this visit, I feel I can cope better with my health problem/ illness than before; with answering options yes, no).

To measure PREMs we used four scales, used in previous analyses of the QUALICOPC data [18]. The scales were developed using ecometric (latent variable) analysis [19]. The four scales represent important aspects of primary care: Doctor-patient communication (example item: The doctor listened carefully to me), accessibility (example item: The doctor took sufficient time), continuity (example item: The doctor knows important information about my medical background), and comprehensiveness of care (example item: The doctor asked about possible other problems besides the one I just came for).

### Selection and measurement of potential case-mix variables

We selected the following potential case-mix variables (see also Box 1 in the results section):

Demographic: age (based on year of birth: What is your year of birth?), sex (Are you male or female?), migrant background; based on country of birth and mother’s country of birth (Where were you born? Where was your mother born?) three categories were constructed: first generation migrant, second generation migrant and non-migrant).

Socioeconomic: household income (Compared to the average income in this country, would you say your household’s income is: below average, average, above average?), education (What is the highest level of education that you achieved? Low, middle, high).

Health status: chronic disease (Do you have a longstanding disease or condition such as high blood pressure, diabetes, depression, asthma or another longstanding condition? Yes, no) and self-rated general health (if the latter is not used as a PROM in itself).

Location of the practice (from GP questionnaire as proxy for the place of living of the patients): How would you characterize the place you are currently practicing? With answering categories: Big (inner)city, suburbs, (small) town, mixed urban–rural, rural.

### Statistical analysis

We applied multilevel regression analysis for each of the PREMs ad PROMs, with three levels:

Level 1: patients, level 2: GPs, level 3: countries. The PREMs we use in this study are composite variables, consisting of several items. They have been constructed in a multilevel latent variable analysis with four levels, the lowest level being the separate items [19]. Fixed effects and random (slope) effects will be assessed. In this case we use linear multilevel analysis. Of the PROMs, we consider self-reported health as a continuous variable and apply linear multilevel analysis; for the enablement item, we applied logistic regression and used pi squared divided by three as approximation of the individual level variance [20].

If random slope effects are statistically significant, this does not mean that they are also relevant. The difference between the variances in the different categories of a potential case-mix adjuster can be very small, but still significant, depending on the shape of the distribution and the number of observations. From the point of view of improvement potential, the difference has to be sufficiently large to warrant action.

For differences in outcomes in the arms of trials, the idea of a minimum clinically important difference (MCID) has been proposed, first suggested in 1989 by Jaeschke et al. [21]. We use this literature to decide whether a certain slope variation coincides with differences between units that are big enough to say that they are meaningful. If there are big differences between units, some units have better outcomes than others and case-mix adjustment is not indicated. The literature on MCID is based on differences between measurements of an outcome, such as a PROM, before and after a treatment. It argues that statistical significance is not a good basis, because it depends on the number of observations.

There are three approaches to finding a MCID (see review by Sedaghat, 2019 [22]):

- the distributional approach which looks at the distribution of the outcome and uses the standard deviation (SD) or the standard error of measurement;
- the anchor approach which uses a judgement of patients whether or not (or to what extent) their health situation has improved after the treatment;
- the consensus approach which assesses what experts/clinicians see as a clinically important difference. The last two approaches are less useful in case of PREMs and PROMs in cross-sectional research and in the absence of specific treatments.

According to reviews [22, 23] a rule of the thumb, based on many different situations, is that a difference between the before and after treatment situation of 0.5*SD, based on the distribution of the outcome before treatment, can be seen as the MCID. This is equivalent to medium effect size. As this is based on the context of treatment interventions, it is not directly applicable to our problem.

In our case we want to assess whether a slope variance is sufficiently large to decide that case-mix adjustment is not indicated. We can do this by comparing the variances in the categories of a potential case-mix adjuster. The difference in outcome of 0.5*SD, as used in before-after measurements in treatment situations, can be translated into a difference between the variances of 0.25*variance, as the SD is the square root of the variance.

### Modelling strategy

Our modelling strategy consists of the following steps:

1. Multilevel analysis to assess the relationship of the potential case-mix adjusters with the PREMs and PROMs. Random effects at the level of the GPs and the countries and fixed effects for the potential case-mix adjusters. We included one independent variable at a time. If no relation, no need to adjust. In this step regression lines are modelled as parallel lines.
2. Multilevel analysis with random effects at the level of the GPs and the countries and random slopes for the potential case-mix adjusters. If the slope variation turns out to be statistically significant and large enough, correction for case-mix is not indicated. This may be an indication that some GPs/countries ‘produce’ better outcomes than others. There may be situations where the overall fixed effect is not significant, but there is still significant variation in one or more of the categories of the independent variable. This also provides information about improvement possibilities. In this step regression lines are allowed to vary.
3. If there is no significant slope variation for an independent variable or the variation is not substantial (although significant), this variable is a case-mix adjuster if the distribution of this variable differs between units. Hence, we then analyse the heterogeneity of the potential case-mix variables between units. We use the potential case-mix variables as dependent variables in a null model. Significant variation at the level of the GPs and/or countries indicates that the composition of the units for this variable differs. In this step only the variances at different levels in the potential case-mix variables are modelled.

### Ethical approval

Ethical approval for the QUALICOPC study was acquired in accordance with the legal requirements in each country [24].

## Results

**Fig. 1:**
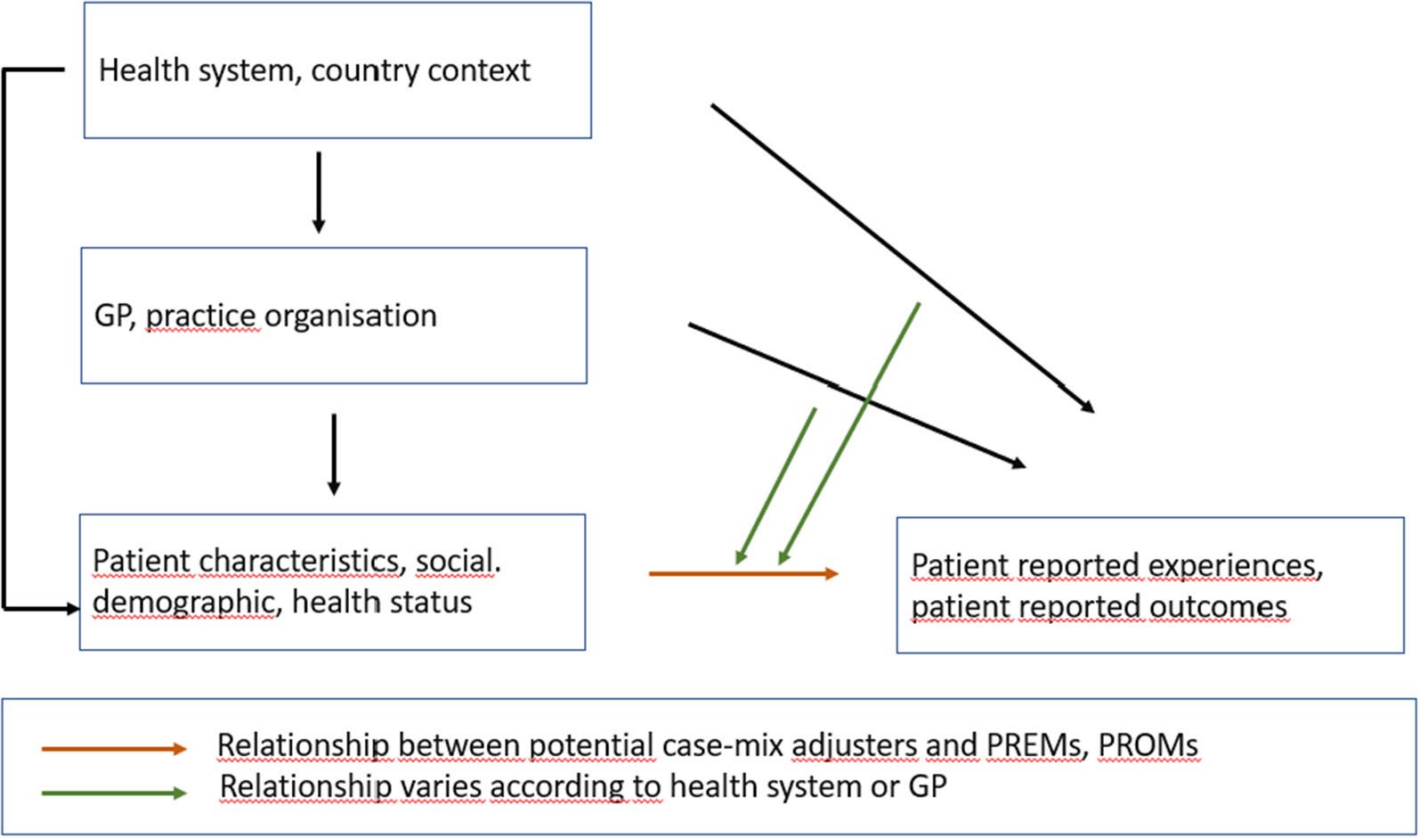
Conceptual model to guide the development of case-mix adjusters

To illustrate the data structure and to develop the case-mix adjusters, we use a conceptual framework, based on the conceptual model of the QUALICOPC study (Fig. 1). This conceptual framework shows that there are three levels involved—health system/country, GP/GPpractice and patients. The health system and GP levels may influence how patients experience care and the outcomes they report. The patient characteristics that are potential case-mix adjusters relate to the PREMs and PROMs (red arrow, main effects of patient characteristics) and these relationships may depend on the health system and GP practice the patients belong to (green arrows, slope variation).

### Selection of potential case-mix adjusters

We have reviewed the literature to find out which variables are commonly used for case-mix adjustment in the context of primary care. We have not found literature about case-mix adjustment in relation to PROMs in primary care. PROMs are usually disease specific and were mostly developed in specialist care. For PREMs we have used literature that was focused on the identification of case-mix adjusters. The results are in Box 1.

**Box 1:** Variables from the literature about case-mix adjustment in PREMs in primary care

*Johnson *et al.,* 2010* [25]:	lll
Outcome variable: PREMs from CAHPS	
Case-mix adjusters: self-reported health, age, education, race/ethnicity, usual language spoken	
*Damman *et al.,* 2011* [11]:	
Outcome variable: PREMs from Consumer Quality index General Practice	
Case-mix adjusters: age, general health status, mental health status, education, sex, and ethnicity	
*Paddison *et al.,* 2012* [7]:	
Outcome variable: PREMs in three domains of primary care: access; interpersonal care; anticipatory care planning, and overall satisfaction with primary care services	
Case-mix adjusters: age, sex, ethnicity, self-reported health, and socio-economic status of residential address	
*Hatfield and Zaslavsky (2017)* [13]:	
Outcome variable: PREMs of process quality from CAHPS	
Case-mix adjusters: self-reported general health status, mental health status, education level	

These potential case-mix adjusters a priori make sense. Most of these variables are in the QUALICOPC dataset and most are also included in the PaRIS patient questionnaire. Exceptions are usual language spoken (not in the PaRIS questionnaire) and mental health status (not in the QUALICOPC dataset). Hence we retain as potential case-mix adjusters: Self-reported general health, having a longstanding disease, patients’ age, sex, level of education, household income, migration status and place of living. One may argue that self-reported general health is not beyond control of the units and therefore should not be used as a case-mix adjuster for PREMs. However, in a cross-sectional study, without a before-measurement of self-reported health, we think it can be considered a potential case-mix adjuster. We report the results of the analyses in a summary table for each PREM and PROM (Tables 1, 2, 3, 4, 5, 6) and provide the details for each combination of the potential case-mix adjusters and the PREMs and PROMs in Additional file 1.

**Table 1:** Summary of the analyses for the dependent variable Communication (PREM), multilevel linear regression

Potential case-mix variable	Fixed effect significant (y/n)	Slope effect GP level important* (y/n)	Slope effect Country level important (y/n)	Case-mix adjustment (y/n)
Self-reported general health	Yes Worse self-reported health → experienced communication worse	No	No	Yes
Longstanding disease	Yes Longstanding disease → experienced communication worse	No	No	Yes
Patient’s age	Yes, Older patients → experienced communication worse	No	No	Yes
Patient’s sex	No	No	No	No
Education	Yes Higher education → experienced communication better	Yes	No	No Importantly more variation at GP level with lower educated
Income	Yes Higher household income → experienced communication better	No	No	Yes
Migrant status	Yes 1st generation migrant → experienced communication worse	Yes	No	No Importantly more variation at GP level with 1st generation migrants
Place of living	No	Yes	No	No No significant fixed effect, but importantly more variation in small towns compared to suburbs and rural areas

*Important means that the difference in variance between categories is more that 0.25*variance in the model with fixed effect and random intercept

**Table 2:** Summary of the analyses for the dependent variable Access (PREM), multilevel linear regression

Potential case-mix variable	Fixed effect significant (y/n)	Slope effect GP level important* (y/n)	Slope effect Country level important (y/n)	Case-mix control (y/n)
Self-reported general health	Yes Worse self-reported health → worse experienced access	No	No	Yes
Longstanding disease	No	No	No	No
Patient’s age	Yes Older than 40 → better experienced access	No	No	Yes
Patient’s sex	No	No	No	No
Education	Yes Higher education → better experienced access	No	No	Yes
Income	Yes Higher income → better experienced access	No	No	Yes
Migrant status	Yes Migrant status → lower experienced access	No	No	Yes
Place of living	Yes Outside big cities → better experienced access	Yes	No	No Importantly more variation big (inner) cities compared to rural areas

^*^Important means that the difference in variance between categories is more that 0.25*variance in the model with fixed effect and random intercept

**Table 3:** Summary of the analyses for the dependent variable Continuity of care (PREM), multilevel linear regression

Potential case-mix variable	Fixed effect significant (y/n)	Slope effect GP level important* (y/n)	Slope effect country level important (y/n)	Case-mix control (y/n)
Self-reported general health	Yes Worse self-reported health → better experience continuity	No	Yes	No Importantly more variation at country level for people with very good self-reported health
Longstanding disease	Yes Longstanding disease → better experienced continuity	No	No	Yes
Patient’s age	Yes Older than 40 → better experienced comtimuity	No	No	Yes
Patient’s sex	Yes Women → better experienced continuity	No	No	Yes
Education	Yes Higher education → worse experienced continuity	No	No	Yes
Income	No	No	No	No
Migrant status	Yes Migrant status → worse experienced continuity	No	No	Yes
Place of living	Yes Outside big cities and suburbs → better experienced continuity	No	No	Yes

^*^Important means that the difference in variance between categories is more that 0.25*variance in the model with fixed effect and random intercept

**Table 4:** Summary of the analyses for the dependent variable Comprehensiveness of care (PREM), multilevel linear regression

Potential case-mix variable	Fixed effect significant (y/n)	Slope effect GP level important* (y/n)	Slope effect country level important (y/n)	Case-mix control (y/n)
Self-reported general health	Yes Worse self-reported health → more experienced comprehensiveness	No	No	Yes
Longstanding disease	Yes Longstanding disease → more experienced comprehensiveness	No	No	Yes
Patient’s age	Yes Older than 40 → better experienced comprehensiveness	No	No	Yes
Patient’s sex	No	No	No	No
Education	Yes Higher education → less experienced comprehensiveness	No	No	Yes
Income	Yes Higher income → less experienced comprehensiveness	No	No	Yes
Migrant status	Yes Second generation migrants → less experienced comprehensiveness	No	No	Yes
Place of living	Yes In mixed urban–rural and rural areas → more experienced comprehensiveness	No	No	Yes

^*^Important means that the difference in variance between categories is more that 0.25*variance in the model with fixed effect and random intercept

**Table 5:** Summary of the analyses for the dependent variable Coping after the consultation (PROM), multilevel logistic regression

Potential case-mix variable	Fixed effect significant (y/n)	Slope effect GP level important* (y/n)	Slope effect country level important (y/n)	Case-mix control (y/n)
Self-reported general health	Yes Poor self-reported health → less able to cope	No	No	Yes
Longstanding disease	No	No	No	No
Patient’s age	Yes Older patients → better able to cope	No	No	Yes
Patient’s sex	Yes Women → better able to cope	No	No	Yes
Education	Yes Higher educated → less able to cope	No	No	Yes
Income	Yes Middle income → better able to cope	No	No	Yes
Migrant status	Yes Second generation migrant → less able to cope	No	No	Yes
Place of living	Yes In mixed urban–rural and rural areas → better able to cope	No	No	Yes

^*^Important means that the difference in variance between categories is more that 0.25*variance in the model with fixed effect and random intercept

**Table 6:** Summary of the analyses for the dependent variable Self-reported health (PROM), multilevel linear regression

Potential case-mix variable	Fixed effect significant (y/n)	Slope effect GP level important* (y/n)	Slope effect country level important (y/n)	Case-mix control (y/n)
Longstanding disease	Yes Longstanding disease → worse self-reported health	No	No	Yes
Patient’s age	Yes Older patients → worse self-reported health	No	No	Yes
Patient’s sex	Yes Women → worse self-reported health	No	No	Yes
Education	Yes Higher educated → better self-reported health	No	No	Yes
Income	Yes Higher income → better self-reported health	No	No	Yes
Migrant status	Yes First generation migrant → worse self-reported health	No	No	Yes
Place of living	Yes In mixed urban–rural and rural areas → worse self-reported health	No	No	Yes

^*^Important means that the difference in variance between categories is more that 0.25*variance in the model with fixed effect and random intercept

### Assessment of the potential case-mix adjusters

The first step in the analysis was to find out whether the potential case-mix variables are actually associated with the PREMs and PROMs. This is reported in the second column of Tables 1, 2, 3, 4, 5, 6. In the third and fourth column we report whether or not the slope effect at GP and/or country level is important, using the criterion that the difference in variance between categories is more than 0.25 times the total variance at all levels in the model with a fixed effect. The final column states whether or not case mix adjustment is indicated according to the steps taken in this study. It turned out that in the dataset used, the distribution of the potential case-mix adjusters always differs between units, judged from the higher level variances that all differ significantly from zero. Therefore, we have not included this in Tables 1, 2, 3, 4, 5, 6; however, the intraclass correlations (ICCs) are included in Additional file 1: table G1. The smallest ICCs at both country and GP level are found for patient’s sex; this means that the sex distribution differs least. The largest ICC at country level is for migrant status; 27.5% of the variation is at country level. The largest ICC at GP level is for age of patients; 20.7% of the variation in patients aged 76 years and over is at the level of the GPs (Additional file 1: table G1).

For patient experienced communication with their GP, the first PREM we consider (Table 1), we find important slope effects for patients’ education, with more variation among GPs in communication as experienced by patients with lower education; for patients’ migration background, with more variation among GPs in communication as experienced by first generation migrants; and patients’ place of living, with more variation among GPs in communication as experienced by patients living in small towns. There is no important variation at country level. For these variables case-mix adjustment is not indicated. According to our criteria, case-mix adjustment is also not indicated for patients’ sex, as there is no significant main effect (see also Additional file 1: tables A1-A8).

Patient reported access (Table 2) only shows an important slope effect for patients’ place of living: there is more variation between GPs in patient reported access among those living in big (inner) city areas, compared to those living in rural areas. Hence, case-mix adjustment is not indicated in this case. According to our criteria, case-mix adjustment is also not indicated for whether or not patients have a longstanding disease and for patients’ sex, as there are no significant main effect (see also Additional file 1: tables B1-B8)﻿.

Continuity of care (Table 3) shows importantly more variation at country level for people with very good self-reported health. According to our criteria, case-mix adjustment is not indicated in this case. The same for household income, as there is no significant main effect and no important slope effects (see also Additional file 1: tables C1-C8)﻿.

The analyses for comprehensiveness of care (Table 4) do not show any important slope effects, either at GP or country level. Case-mix adjustment is not indicated for patients’ sex, as there is no significant main effect (see also Additional file 1: tables D1-D8)﻿.

With the first PROM, the enablement question (Table 5), we did not find any important slope effects, either at GP or country level. Case-mix adjustment is not indicated for whether or not patients have a longstanding disease, as there is no significant main effect (see also Additional file 1: tables E1-E8)﻿.

With the second PROM, self-reported health (Table 6), there is no important slope variation at either level for any of the potential case-mix variables. Given that the main effects are all significant, case-mix adjustment is indicated (see also Additional file 1: tables F1-F7)﻿.

## Discussion

### Summary of the results

We developed a multilevel approach to case-mix adjustment, that takes into account both the fixed effect of potential case-mix adjusters and whether the effect of a variable differs between units, indicated by a varying slope. To assess whether the slope variation is not just significant but also of an important size we borrowed the empirical generalisation from research into MCID in the area of treatment trials and translated this to variances. We applied our approach to the international comparison of PROMs and PREMs in primary care, using data from the QUALICOPC study. Our approach represents an improvement over standard methods of case mix adjustment that often only use the strength of the relationship with an outcome variable and the heterogeneity of the distribution of the potential case-mix variable over the units that are compared.

It turned out that in the dataset used, the distributions of all potential case-mix adjusters differed between countries and GPs in all cases. Case-mix adjustment is not indicated when there is important variation in the relationship between a potential case-mix adjuster and a PREM or PROM. In this case the variation may point to potential for improvement. This is the case with education, ethnicity and place of living, and patient experienced communication, place of living and access to care, and self-rated health and continuity of care. There is also no need for case-mix adjustment in our analysis when there is no fixed effect. This is the case with sex of the patient and three of the PREMs, household income and continuity of care, and having a chronic disease and access to care (Table 7). From this overview table we conclude that the need for case-mix adjustment differs between dimensions of PREMs and PROMs; with the experienced GP-patient communication adjustment is least often indicated. It also differs between patient characteristics; for the age of patients case-mix adjustment was indicated for all PREMs and PROMs considered, while with place of living of patients we found important slope variation with two different PREMs.

**Table 7:** Overview of potential case-mix adjusters and whether or not adjustment is indicated

	Communi-cation	Access	Continuity	Comprehen-siveness	Self-reported health	Enable-ment
Age	Yes	Yes	Yes	Yes	Yes	Yes
Sex	No fixed effect	No fixed effect	Yes	No fixed effect	Yes	Yes
Education	No; slope effect important	Yes	Yes	Yes	Yes	Yes
Household income	Yes	Yes	No fixed effect	Yes	Yes	Yes
Ethnicity	No; slope effect important	Yes	Yes	Yes	Yes	Yes
Place of living	No; slope effect important	No; slope effect important	Yes	Yes	Yes	Yes
Self-rated health	Yes	Yes	No; slope effect important	Yes	-	Yes
Longstan-ding disease	Yes	No fixed effect	Yes	Yes	Yes	No fixed effect

### Challenges for the application of case-mix adjusters for international comparison

PROMs and PREMs are mainly used to assess and improve the performance of health care providers, such as primary care practices or hospitals within a country. In international comparisons, average PREMs and PROMs are compared between countries. This not only requires fairness of comparison between providers, but also between countries. In the multilevel analyses we applied, both the level of providers within countries and the level of the countries are taken into account. There can be meaningful differences in PREMs and PROMs between providers in their performance for different patient categories as well as between countries. We found several cases of important variation between GPs and only one case in which the important variation was between countries. This was the case with the relationship between self-rated health and the PREM continuity of care. In general, people with worse self-reported health experienced better continuity of care (and we should add that they are often in a better position to assess continuity of care). At the same time, we found importantly more variation at country level for people with very good self-reported health, compared to those with poor self-rated health.

When we apply case-mix adjustment to reach fair comparisons between providers and/or countries, we implicitly assume that it is meaningful to apply an average population to all providers and/or countries. This will usually not be a problem when comparing providers within countries and when comparing countries that do not differ much in their patient population (although there should be heterogeneity in the distribution to qualify as a potential case-mix adjuster). However, when comparing countries with large differences in the composition of their population, an average population intuitively makes less sense. The largest difference between countries in distribution of patient groups is for patients with a migration background. In this case, applying a standard population could be debated.

### Measurement equivalence

The source of performance differences between and within countries may not only be ‘real’ differences in performance of care providers. The measurement of PREMs and PROMs may not be the same in different countries [26]. For example, cultural differences between countries and patient groups may show up as performance differences between units.

In case of the use of surveys to assess outcomes (as in PREMs and PROMs), the relationship between a potential case-mix variable and an outcome may be influenced by varying expectations [27, 28] and by response tendencies, such as social desirability [3]. In case of a composite measure as outcome variable (a scale to measure a PREM or PROM), response tendencies may differ between items that form the scale. This is usually called differential item functioning and is addressed by improving the scale rather than by case-mix controls.

When comparing countries, we can subsume expectations and response tendencies under cultural differences. Varying expectations and response tendencies are particularly important when comparing over patient groups [29] or over countries, as in this paper. This goes into the question as to *why* patient groups differ in their experiences and outcomes and whether these may differ even when the ‘objective’ situation in terms of care provision is the same. For example, older patients may be more inclined to report positive experiences because their expectations are lower. This is relevant in the context of case-mix controls when for various reasons there are differences between units in the response tendency or expectations of particular patient groups. It is difficult to imagine how a statistical analysis alone is able to distinguish between a varying response tendency or a variation that indicates a difference that is under the influence of providers.

In our view, additional information would be needed to make the distinction. A possibility is to measure differences in expectations. This has been done in the Dutch Quality of care through the patients’ eyes (QUOTE)-questionnaires [30], the Consumer Quality index [31], and in the QUALICOPC survey [1] by measuring ‘instrumental values’ about health service provision. In these surveys instrumental values were measured by asking how important respondents find a certain aspect of care provision [18, 32, 33]. This is particularly important for international comparisons where cultural differences and differences in the structure of health care systems may lead to different expectations and instrumental values. We have not explored this in this paper. The main reason is that measurement of instrumental values is not planned for the PaRIS survey.

Response tendencies, such as the tendency to give socially desirable answers, may differ between patient groups and between countries. We did not find literature that assesses tendencies in answering PREMs and PROMs survey questions in different countries or patient groups. The literature on international comparisons of answering tendencies in general shows differences between countries [34, 35]. PREMs and PROMs are perhaps not subjects where social desirability plays a heavy role, when anonymity is guaranteed and results are not used to publish rankings of providers. However, deference to doctors may influence how people answer to PREMs and this may differ between countries.

Answering tendencies may be measured directly in surveys through e.g. social desirability scales, or derived from the frequency of answers given, e.g. the tendency to answer yes or no, or to use extreme categories. To take differences in answering tendencies between countries into account, they do not have to be measured in a PREMs or PROMs survey. For example, social desirability tendencies may also be measured independently in other surveys and used in the interpretation of a PREM/PROM survey by linking data at the level of patient groups or countries. However, we have not found international databases that contain measurements of social desirability that could be used as variables in our analysis.

### Is performance improvement a realistic aim?

At the background of performance measurement and case-mix adjustment is the idea that some differences in performance would require improvement action/policies. However, we do not take the affordability of producing good outcomes/processes into account. Primary care can only be made accountable for what is achievable within a given health service delivery system [36]. Accessibility differs between GP practices, depending on their location and it is also under the influence of care providers and policy-makers, but perhaps we cannot expect all countries to be able to invest equally in access to care. As an example, in our analysis access to care outside big (inner) cities is experienced as better and there is importantly more variation between GPs in big (inner) cities compared to rural areas in patient experienced access. The question is then whether GPs/practices can be held accountable for this or whether it is something else in urban areas that causes the patients’ experiences. To address this question would require an additional and different type of analysis. At country level, an analysis of the inputs (money, human resources) in relation to the outputs (case-mix adjusted PREMs and PROMs) can be done using Data Envelopment Analysis (DEA), such as for example used in the World Health Report 2000 [37].

### Limitations

The steps to identify case-mix adjusters do not always apply. There is at least one exception: If—for example—all providers/all countries discriminate against migrants in access to care to the same extent, it could still be under the influence of providers or countries to improve access for these groups.

Our criterion to assess the importance of slope variation seems to work well with PREMs and PROMs that can be considered as continuous variables. However, with binary PREMs or PROMs, such as the enablement question in this study, the approximation of individual level variance is always much larger than the variance at the other levels. Hence, single questions with answering categories that cannot be considered continuous, should be avoided as much as possible.

The criterion to assess the importance of the slope variation is not absolute [23] and the cut-off point for a MCID is not uncontested. Wyoane-Hune et al. propose 1/3*SD_baseline_ (in combination with an anchor approach) [38]. Our approach is therefore more conservative, which makes sense in view of the fact that we are not evaluating clinical interventions but evaluations of a broad service, in this case primary care. However, sensitivity analysis could be used to assess the impact of alternative cut-off points on the variables selected for case-mix adjustment and, ultimately, on the case-mix adjusted comparisons.

We have used a large and rich dataset on patients’ experiences of care. Patient-reported outcomes were less extensively measured, with only a generic measurement of self-rated health and one item on enablement. The enablement item is relevant as a PROM in primary care. The extent to which people feel they can cope better than before seems particularly relevant in the context of the growing number of people living with chronic diseases and what (primary) health care may achieve for them. So although this PROM is not the typical outcome, as used in clinical studies, it may be argued that in primary it is the ultimate outcome for many patients. Moreover, the patient population in primary care is typically unselected and many consultations are single consultations. This differs fundamentally from, e.g., the situation where planned interventions are evaluated with a PROM before and after the intervention.

Although the data set is somewhat old, this does not affect the development of the approach; only substantive conclusions in terms of improvement needs. By now GP care in the countries included in the QUALICOPC study may have improved and differences between GPs may have declined. Our focus was on the development of the approach. The results of our analysis cannot be generalised to the current situation and to other samples of countries. We have analysed the most important potential case-mix adjusters; however, there may be unmeasured case-mix variables, e.g., relating to specific diseases.

The QUALICOPC data, used in this secondary analysis, have their limitations (as described in the separate publications). The study only evaluated primary care through data collected among GPs and their patients, excluding other providers of primary care. The study was cross-sectional; hence, no changes in PREMs and PROMs at aggregate or individual level could be analysed. Finally, selective non-response may have led to bias, although the participating GPs were representative of the populations by age and sex.

## Conclusion

Our approach can be used to guide decisions about whether or not patient characteristics should be used to adjust for case-mix in an international primary care study, next to theoretical and practical considerations. The criterion of a difference between variances of 0.5*variance, borrowed from the literature on MCID (and equivalent to 0.25*SD), was applied, coinciding with a medium effect size. This works well for continuous PREMs and PROMs. Without additional information, it is impossible to decide whether important slope variation is the result of differences in performance between general practices or countries, or of cultural differences, and to what extent differences in performance are within control of general practices or countries. In the end, the decisions to adjust for case-mix, to decide whether or not a difference in slope variance is deemed important, depends on the research questions and the policy context of the study.

### Supplementary Information

**Additional file 1**. Supplementary Tables.

## Data Availability

The datasets used and/or analysed during the current study are available from the corresponding author on reasonable request.

## Abbreviations

CAHPS: Consumer Assessment of Healthcare Providers and Systems
DEA: Data envelopment analysis
GP: General practitioner
ICC: Intraclass correlation
MCID: Minimum clinically important difference
OECD: Organisation for Economic Cooperation and Development
PaRIS: Patient reported indicator survey
PREMs: Patient reported experience measures
PROMs: Patient reported outcome measures
QUALICOPC: Quality and costs of primary care in Europe
QUOTE: Quality of care through the patients’ eyes
SD: Standard deviation
UK: United Kingdom
