# Supplementary material for: Case-mix adjustments for patient reported experience and outcome measures in primary care: an empirical approach to identify patient characteristics as case-mix adjusters based on a secondary analysis of an international survey among patients and their general practitioners in 34 countries
Source: J Patient Rep Outcomes. 2023 Dec 4;7:127. doi: 10.1186/s41687-023-00667-8 (PMC10695892; doi:10.1186/s41687-023-00667-8)
Supplement: Supplementary file 1 — Additional file 1. Supplementary Tables. [file 41687_2023_667_MOESM1_ESM.docx]

Additional files

Detailed results per variable

Table A1: Dependent variable Communication (PREM), independent variable Self-reported general health, multilevel linear regression

| *Random* | *intercept* |  |  |  |  |
| --- | --- | --- | --- | --- | --- |
| *Fixed effects* | Coefficient (p-value) |  |  |  |  |
| Intercept | 0.964 |  |  |  |  |
| Very good (ref) | - |  |  |  |  |
| Good | -0.00022 (0.65) |  |  |  |  |
| Fair | -0.00174 (<0.001) |  |  |  |  |
| Poor | -0.00367 (<0.001) |  |  |  |  |
| *Random effects* |  | *Random*  Very good | *Slope*  Good | *Model*  Fair | *Variances*  Poor |
| Individual variance | 0.0012 | 0.0009 | 0.0010 | 0.0014 | 0.0019 |
| GP variance | 0.000835 | 0.0007 | 0.0009 | 0.0009 | 0.001 |
| Country variance | 0.000286 | 0.00027 | 0.00028 | 0.0003 | 0.00043 |
| Total variance | 0.00232 |  |  |  |  |
| 0.25*variance | 0.00058 |  |  |  |  |

Table A2: Dependent variable Communication (PREM), independent variable Having a longstanding disease, multilevel linear regression

| *Random* | *Intercept* |  |  |
| --- | --- | --- | --- |
| *Fixed effects* | Coefficient (p-value) |  |  |
| Intercept | 0.963 |  |  |
| No (ref) | - |  |  |
| Yes | -0.0013 (<0.001) |  |  |
| *Random effects* |  | *Random slope*  No | *model variances*  Yes |
| Individual variance | 0.0012 | 0.001 | 0.001 |
| GP variance | 0.000836 | 0.0008 | 0.0009 |
| Country variance | 0.000288 | 0.00028 | 0.00031 |
| Total variance | 0.00232 |  |  |
| 0.25*variance | 0.00058 |  |  |

Table A3: Dependent variable Communication (PREM), independent variable Patient’s age, multilevel linear regression

| *Random* | *Intercept* |  |  |  |  |
| --- | --- | --- | --- | --- | --- |
| *Fixed effects* | Coefficient (p-value) |  |  |  |  |
| Intercept | 0.963 |  |  |  |  |
| 18-40 (ref) | - |  |  |  |  |
| 41-60 | -0.00084 (0.03) |  |  |  |  |
| 61-75 | -0.0025 (<0.001) |  |  |  |  |
| 76 and older | -0.0027  (<0.001) |  |  |  |  |
| *Random effects* |  | *Random slope*  18-40 | *model variances*  41-60 | 61-75 | 76 and older |
| Individual variance | 0.0012 | 0.0011 | 0.0012 | 0.0012 | 0.0012 |
| GP variance | 0.000838 | 0.0008 | 0.0008 | 0.0011 | 0.0009 |
| Country variance | 0.000287 | 0.0003 | 0.0003 | 0.0003 | 0.0004 |
| Total variance | 0.00232 |  |  |  |  |
| 0.25*variance | 0.00058 |  |  |  |  |

Table A4: Dependent variable Communication (PREM), independent variable Patient’s sex, multilevel linear regression

| *Random* | *Intercept* |  |  |
| --- | --- | --- | --- |
| *Fixed effects* | Coefficient (p-value) |  |  |
| Intercept | 0.963 |  |  |
| Male (ref) | - |  |  |
| Female | 0.00039 (0.227) |  |  |
| *Random effects* |  | *Random slope*  Male | *model variances*  Female |
| Individual variance | 0.0012 | 0.0013 | 0.0012 |
| GP variance | 0.000836 | 0.00091 | 0.00082 |
| Country variance | 0.000288 | 0.00029 | 0.00028 |
| Total variance | 0.00232 |  |  |
| 0.25*variance | 0.00058 |  |  |

Table A5: Dependent variable Communication (PREM), independent variable Patient’s education, multilevel linear regression

| *Random* | *Intercept* |  |  |  |
| --- | --- | --- | --- | --- |
| *Fixed effects* | Coefficient (p-value) |  |  |  |
| Intercept | 0.963 |  |  |  |
| Low (ref) | - |  |  |  |
| Middle | 0.00157 (<0.001) |  |  |  |
| High | 0.00378 (<0.001) |  |  |  |
| *Random effects* |  | *Random slope*  Low | *model variances*  Middle | High |
| Individual variance | 0.0012 | 0.0013 | 0.0013 | 0.009 |
| GP variance | 0.000836 | 0.00119* | 0.00093 | 0.00058* |
| Country variance | 0.000288 | 0.00034 | 0.00029 | 0.00023 |
| Total variance | 0.00232 | ICC_GP_=42% | ICC_GP_=37% | ICC_GP_=34% |
| 0.25*variance | 0.00058 |  |  |  |

*Important difference in variance

Table A6: Dependent variable Communication (PREM), independent variable Household income, multilevel linear regression

| *Random* | *Intercept* |  |  |  |
| --- | --- | --- | --- | --- |
| *Fixed effects* | Coefficient (p-value) |  |  |  |
| Intercept | 0.963 |  |  |  |
| Low (ref) | - |  |  |  |
| Middle | 0.00152 (<0.001) |  |  |  |
| High | 0.00259 (<0.001) |  |  |  |
| *Random effects* |  | *Random slope*  Low | *model variances*  Middle | High |
| Individual variance | 0.0012 | 0.0014 | 0.0011 | 0.0008 |
| GP variance | 0.000835 | 0.0011 | 0.0007 | 0.00069 |
| Country variance | 0.000286 | 0.00031 | 0.00028 | 0.00026 |
| Total variance | 0.00232 |  |  |  |
| 0.25*variance | 0.00058 |  |  |  |

Table A7: Dependent variable Communication (PREM), independent variable Migrant status, multilevel linear regression

| *Random* | *Intercept* |  |  |  |
| --- | --- | --- | --- | --- |
| *Fixed effects* | Coefficient (p-value) |  |  |  |
| Intercept | 0.963 |  |  |  |
| Born in this country (ref) | - |  |  |  |
| First generation migrant | -0.00288 (<0.001) |  |  |  |
| Second generation migrant | -0.00092 (0.25) |  |  |  |
| *Random effects* |  | *Random slope*  Born in this country | *model variances*  First generation migrant | Second generation migrant |
| Individual variance | 0.0012 | 0.0012 | 0.0013 | 0.0009 |
| GP variance | 0.000835 | 0.00082 | 0.00116* | 0.00029* |
| Country variance | 0.000286 | 0.00029 | 0.00032 | 0.00033 |
| Total variance | 0.00232 | ICC_GP_=35% | ICC_GP_=42% | ICC_GP_=20% |
| 0.25*variance | 0.00058 |  |  |  |

*Important difference in variance

Table A8: Dependent variable Communication (PREM), independent variable Place of living, multilevel linear regression

| *Random* | *Intercept* |  |  |  |  |  |
| --- | --- | --- | --- | --- | --- | --- |
| *Fixed effects* | Coefficient (p-value) |  |  |  |  |  |
| Intercept | 0.963 |  |  |  |  |  |
| Big (inner) city (ref) | - |  |  |  |  |  |
| Suburbs | 0.00022 (0.88) |  |  |  |  |  |
| (Small) Towns | -0.00047 (0.67) |  |  |  |  |  |
| Mixed urban-rural | 0.0012 (0.34) |  |  |  |  |  |
| Rural | 0.0023 (0.07) |  |  |  |  |  |
| *Random effects* |  | *Random*  Big (inner) city | *slope*  Suburbs | *model*  (Small) towns | *variances*  Mixed urban-rural | Rural |
| Individual variance | 0.0012 | 0.0012 | 0.0011 | 0.0014 | 0.0012 | 0.00088 |
| GP variance | 0.000836 | 0.00091 | 0.00055* | 0.0012* | 0.00063 | 0.00054* |
| Country variance | 0.000284 | 0.00028 | 0.00027 | 0.00031 | 0.00036 | 0.00027 |
| Total variance | 0.00232 | ICC_GP_=37% | ICC_GP_=29% | ICC_GP_=40% | ICC_GP_=29% | ICC_GP_=32% |
| 0.25*variance | 0.00058 |  |  |  |  |  |

*Important difference in variance

Table B1: Dependent variable Access (PREM), independent variable Self-reported general health,

multilevel linear regression

| *Random* | *intercept* |  |  |  |  |
| --- | --- | --- | --- | --- | --- |
| *Fixed effects* | Coefficient (p-value) |  |  |  |  |
| Intercept | 0.850 |  |  |  |  |
| Very good (ref) | - |  |  |  |  |
| Good | -0.0004 (0.126) |  |  |  |  |
| Fair | -0.0013 (<0.001) |  |  |  |  |
| Poor | -0.0034  (<0.001) |  |  |  |  |
| *Random effects* |  | *Random*  Very good | *Slope*  Good | *Model*  Fair | *Variances*  Poor |
| Individual variance | 0.0003 | 0.00031 | 0.00033 | 0.00035 | 0.00045 |
| GP variance | 0.0047 | 0.0041 | 0.0046 | 0.0047 | 0.0050 |
| Country variance | 0.0037 | 0.0034 | 0.0036 | 0.0037 | 0.0034 |
| Total variance | 0.0087 |  |  |  |  |
| 0.25*variance | 0.0022 |  |  |  |  |

Table B2: Dependent variable Access (PREM), independent variable Having a longstanding disease, multilevel linear regression

| *Random* | *Intercept* |  |  |
| --- | --- | --- | --- |
| *Fixed effects* | Coefficient (p-value) |  |  |
| Intercept | 0.850 |  |  |
| No (ref) | - |  |  |
| Yes | -0.0001 (0.649) |  |  |
| *Random effects* |  | *Random slope*  No | *model variances*  Yes |
| Individual variance | 0.00034 | 0.00035 | 0.00033 |
| GP variance | 0.0047 | 0.0047 | 0.0046 |
| Country variance | 0.0037 | 0.0037 | 0.0036 |
| Total variance | 0.0087 |  |  |
| 0.25*variance | 0.0022 |  |  |

Table B3: Dependent variable Access (PREM), independent variable Patient’s age, multilevel linear regression

| *Random* | *Intercept* |  |  |  |  |
| --- | --- | --- | --- | --- | --- |
| *Fixed effects* | Coefficient (p-value) |  |  |  |  |
| Intercept | 0.894 |  |  |  |  |
| 18-40 (ref) | - |  |  |  |  |
| 41-60 | 0.0001 (p<0.001) |  |  |  |  |
| 61-75 | 0.0015 (p<0.001) |  |  |  |  |
| 76 and older | 0.0016 (p<0.001) |  |  |  |  |
| *Random effects* |  | *Random slope*  18-40 | *model variances*  41-60 | 61-75 | 76 and older |
| Individual variance | 0.00034 | 0.00039 | 0.00034 | 0.00029 | 0.00025 |
| GP variance | 0.0047 | 0.0049 | 0.0047 | 0.0045 | 0.0037 |
| Country variance | 0.0037 | 0.0037 | 0.0037 | 0.0036 | 0.0039 |
| Total variance | 0.0087 |  |  |  |  |
| 0.25*variance | 0.0022 |  |  |  |  |

Table B4: Dependent variable Access (PREM), independent variable Patient’s sex, multilevel linear regression

| *Random* | *Intercept* |  |  |
| --- | --- | --- | --- |
| *Fixed effects* | Coefficient (p-value) |  |  |
| Intercept | 0.850 |  |  |
| Male (ref) | - |  |  |
| Female | 0.0003 (0.153) |  |  |
| *Random effects* |  | *Random slope*  Male | *model variances*  Female |
| Individual variance | 0.00034 | 0.00036 | 0.00033 |
| GP variance | 0.0047 | 0.0048 | 0.0047 |
| Country variance | 0.0037 | 0.0037 | 0.0037 |
| Total variance | 0.0087 |  |  |
| 0.25*variance | 0.0022 |  |  |

Table B5: Dependent variable Access (PREM), independent variable Patient’s education, multilevel linear regression

| *Random* | *Intercept* |  |  |  |
| --- | --- | --- | --- | --- |
| *Fixed effects* | Coefficient (p-value) |  |  |  |
| Intercept | 0.850 |  |  |  |
| Low (ref) | - |  |  |  |
| Middle | 0.0011 (<0.001) |  |  |  |
| High | 0.0016 (<0.001) |  |  |  |
| *Random effects* |  | *Random slope*  Low | *model variances*  Middle | High |
| Individual variance | 0.00034 | 0.00035 | 0.00034 | 0.00032 |
| GP variance | 0.0047 | 0.0047 | 0.0048 | 0.0046 |
| Country variance | 0.0037 | 0.0036 | 0.0036 | 0.0037 |
| Total variance | 0.0087 |  |  |  |
| 0.25*variance | 0.0022 |  |  |  |

Table B6: Dependent variable Access (PREM), independent variable Household income, multilevel linear regression

| *Random* | *Intercept* |  |  |  |
| --- | --- | --- | --- | --- |
| *Fixed effects* | Coefficient (p-value) |  |  |  |
| Intercept | 0.850 |  |  |  |
| Low (ref) | - |  |  |  |
| Middle | 0.0024 (<0.001) |  |  |  |
| High | 0.0024 (<0.001) |  |  |  |
| *Random effects* |  | *Random slope*  Low | *model variances*  Middle | High |
| Individual variance | 0.00034 | 0.00044 | 0.00030 | 0.00026 |
| GP variance | 0.0047 | 0.0049 | 0.0046 | 0.0042 |
| Country variance | 0.0037 | 0.0039 | 0.0036 | 0.0032 |
| Total variance | 0.0087 |  |  |  |
| 0.25*variance | 0.0022 |  |  |  |

Table B7: Dependent variable Access (PREM), independent variable Migrant status, multilevel linear regression

| *Random* | *Intercept* |  |  |  |
| --- | --- | --- | --- | --- |
| *Fixed effects* | Coefficient (p-value) |  |  |  |
| Intercept | 0.850 |  |  |  |
| Born in this country (ref) | - |  |  |  |
| First generation migrant | -0.0042 (<0.001) |  |  |  |
| Second generation migrant | -0.0017 (<0.001) |  |  |  |
| *Random effects* |  | *Random slope*  Born in this country | *model variances*  First generation migrant | Second generation migrant |
| Individual variance | 0.00034 | 0.00035 | 0.00031 | 0.00023 |
| GP variance | 0.0047 | 0.0047 | 0.0039 | 0.0035 |
| Country variance | 0.0037 | 0.0037 | 0.0036 | 0.0039 |
| Total variance | 0.0087 |  |  |  |
| 0.25*variance | 0.0022 |  |  |  |

Table B8: Dependent variable Access (PREM), independent variable Place of living, multilevel linear regression

| *Random* | *Intercept* |  |  |  |  |  |
| --- | --- | --- | --- | --- | --- | --- |
| *Fixed effects* | Coefficient (p-value) |  |  |  |  |  |
| Intercept | 0.850 |  |  |  |  |  |
| Big (inner) city (ref) | - |  |  |  |  |  |
| Suburbs | 0.004 (0.182) |  |  |  |  |  |
| (Small) Towns | 0.0065 (0.007) |  |  |  |  |  |
| Mixed urban-rural | 0.014 (<0.001) |  |  |  |  |  |
| Rural | 0.024 (<0.001) |  |  |  |  |  |
| *Random effects* |  | *Random slope*  Big (inner) city | *model variances*  Suburbs | (Small) towns | Mixed urban-rural | Rural |
| Individual variance | 0.00035 | 0.00040 | 0.00031 | 0.00039 | 0.00029 | 0.00024 |
| GP variance | 0.0046 | 0.0055* | 0.0036 | 0.0052 | 0.0035 | 0.0034* |
| Country variance | 0.0034 | 0.0038 | 0.0038 | 0.0028 | 0.0032 | 0.0025 |
| Total variance | 0.00835 | ICC_GP_=57% | ICC_GP_=47% | ICC_GP_=62% | ICC_GP_=50% | ICC_GP_=57% |
| 0.25*variance | 0.0021 |  |  |  |  |  |

*Important difference in variance

Table C1: Dependent variable Continuity (PREM), independent variable Self-reported general health, multilevel linear regression

| *Random* | *intercept* |  |  |  |  |
| --- | --- | --- | --- | --- | --- |
| *Fixed effects* | Coefficient (p-value) |  |  |  |  |
| Intercept | 0.886 |  |  |  |  |
| Very good (ref) | - |  |  |  |  |
| Good | 0.013(<0.001) |  |  |  |  |
| Fair | 0.023 (<0.001) |  |  |  |  |
| Poor | 0.031 (<0.001) |  |  |  |  |
| *Random effects* |  | *Random*  Very good | *Slope*  Good | *Model*  Fair | *Variances*  Poor |
| Individual variance | 0.0106 | 0.0013 | 0.0010 | 0.0085 | 0.0011 |
| GP variance | 0.0073 | 0.0092 | 0.0082 | 0.0074 | 0.0054 |
| Country variance | 0.0105 | 0.0151* | 0.0106 | 0.0088 | 0.0050* |
| Total variance | 0.0284 | ICC_Country_=40% | ICC_Country_=37% | ICC_Country_=36% | ICC_Country_=24% |
| 0.25*variance | 0.0071 |  |  |  |  |

*Important difference in variance

Table C2: Dependent variable Continuity (PREM), independent variable Having a longstanding disease, multilevel linear regression

| *Random* | *Intercept* |  |  |
| --- | --- | --- | --- |
| *Fixed effects* | Coefficient (p-value) |  |  |
| Intercept | 0.902 |  |  |
| No (ref) | - |  |  |
| Yes | 0.0234 (<0.001) |  |  |
| *Random effects* |  | *Random slope*  No | *model variances*  Yes |
| Individual variance | 0.0106 | 0.0127 | 0.0077 |
| GP variance | 0.0073 | 0.009 | 0.0057 |
| Country variance | 0.0103 | 0.0136 | 0.0071 |
| Total variance | 0.0282 |  |  |
| 0.25*variance | 0.007 |  |  |

Table C3: Dependent variable Continuity (PREM), independent variable Patient’s age, multilevel linear regression

| *Random* | *Intercept* |  |  |  |  |
| --- | --- | --- | --- | --- | --- |
| *Fixed effects* | Coefficient (p-value) |  |  |  |  |
| Intercept | 0.885 |  |  |  |  |
| 18-40 (ref) | - |  |  |  |  |
| 41-60 | 0.019 (p<0.001) |  |  |  |  |
| 61-75 | 0.030 (p<0.001) |  |  |  |  |
| 76 and older | 0.033 (p<0.001) |  |  |  |  |
| *Random effects* |  | *Random slope*  18-40 | *model variances*  41-60 | 61-75 | 76 and older |
| Individual variance | 0.0106 | 0.014 | 0.0099 | 0.0075 | 0.0046 |
| GP variance | 0.0072 | 0.0095 | 0.0073 | 0.0050 | 0.0052 |
| Country variance | 0.0105 | 0.0153 | 0.0105 | 0.0085 | 0.0082 |
| Total variance | 0.0283 |  |  |  |  |
| 0.25*variance | 0.0071 |  |  |  |  |

Table C4: Dependent variable Continuity (PREM), independent variable Patient’s sex, multilevel linear regression

| *Random* | *Intercept* |  |  |
| --- | --- | --- | --- |
| *Fixed effects* | Coefficient (p-value) |  |  |
| Intercept | 0.902 |  |  |
| Male (ref) | - |  |  |
| Female | 0.0038 (<0.001) |  |  |
| *Random effects* |  | *Random slope*  Male | *model variances*  Female |
| Individual variance | 0.0107 | 0.0118 | 0.0098 |
| GP variance | 0.0074 | 0.0079 | 0.0074 |
| Country variance | 0.0105 | 0.0011 | 0.0011 |
| Total variance | 0.0286 |  |  |
| 0.25*variance | 0.0071 |  |  |

Table C5: Dependent variable Continuity (PREM), independent variable Patient’s education, multilevel linear regression

| *Random* | *Intercept* |  |  |  |
| --- | --- | --- | --- | --- |
| *Fixed effects* | Coefficient (p-value) |  |  |  |
| Intercept | 0.902 |  |  |  |
| Low (ref) | - |  |  |  |
| Middle | -0.0080 (<0.001) |  |  |  |
| High | -0.0126 (<0.001) |  |  |  |
| *Random effects* |  | *Random slope*  Low | *model variances*  Middle | High |
| Individual variance | 0.0107 | 0.0096 | 0.011 | 0.010 |
| GP variance | 0.0073 | 0.0073 | 0.0077 | 0.0079 |
| Country variance | 0.0105 | 0.0083 | 0.0107 | 0.0125 |
| Total variance | 0.0285 |  |  |  |
| 0.25*variance | 0.0071 |  |  |  |

Table C6: Dependent variable Continuity (PREM), independent variable Household income, multilevel linear regression

| *Random* | *Intercept* |  |  |  |
| --- | --- | --- | --- | --- |
| *Fixed effects* | Coefficient (p-value) |  |  |  |
| Intercept | 0.903 |  |  |  |
| Low (ref) | - |  |  |  |
| Middle | -0.002 (0.07) |  |  |  |
| High | -0.0021 (0.21) |  |  |  |
| *Random effects* |  | *Random slope*  Low | *model variances*  Middle | High |
| Individual variance | 0.0107 | 0.0108 | 0.0109 | 0.008 |
| GP variance | 0.0074 | 0.0077 | 0.0073 | 0.0061 |
| Country variance | 0.0105 | 0.0096 | 0.0111 | 0.0088 |
| Total variance | 0.0286 |  |  |  |
| 0.25*variance | 0.0071 |  |  |  |

Table C7: Dependent variable Continuity (PREM), independent variable Migrant status, multilevel linear regression

| *Random* | *Intercept* |  |  |  |
| --- | --- | --- | --- | --- |
| *Fixed effects* | Coefficient (p-value) |  |  |  |
| Intercept | 0.903 |  |  |  |
| Born in this country (ref) | - |  |  |  |
| First generation migrant | -0.0088 (<0.001) |  |  |  |
| Second generation migrant | -0.0071 (0.003) |  |  |  |
| *Random effects* |  | *Random slope*  Born in this country | *model variances*  First generation migrant | Second generation migrant |
| Individual variance | 0.0107 | 0.0109 | 0.0086 | 0.0067 |
| GP variance | 0.0073 | 0.0074 | 0.0048 | 0.0067 |
| Country variance | 0.0106 | 0.0104 | 0.0147 | 0.0141 |
| Total variance | 0.0286 |  |  |  |
| 0.25*variance | 0.0071 |  |  |  |

Table C8: Dependent variable Continuity (PREM), independent variable Place of living, multilevel linear regression

| *Random* | *Intercept* |  |  |  |  |  |
| --- | --- | --- | --- | --- | --- | --- |
| *Fixed effects* | Coefficient (p-value) |  |  |  |  |  |
| Intercept | 0.89 |  |  |  |  |  |
| Big (inner) city (ref) | - |  |  |  |  |  |
| Suburbs | 0.0067 (0.10) |  |  |  |  |  |
| (Small) Towns | 0.0139  (<0.001) |  |  |  |  |  |
| Mixed urban-rural | 0.020 (<0.001) |  |  |  |  |  |
| Rural | 0.0278 (<0.001) |  |  |  |  |  |
| *Random effects* |  | *Random*  Big (inner) city | *slope*  Suburbs | *model*  (Small) towns | *variances*  Mixed urban-rural | Rural |
| Individual variance | 0.0107 | 0.0125 | 0.0107 | 0.0096 | 0.0099 | 0.0093 |
| GP variance | 0.0073 | 0.0089 | 0.0085 | 0.0062 | 0.0055 | 0.0053 |
| Country variance | 0.0103 | 0.0086 | 0.0103 | 0.0056 | 0.0054 | 0.0121 |
| Total variance | 0.0283 |  |  |  |  |  |
| 0.25*variance | 0.0071 |  |  |  |  |  |

Table D1: Dependent variable Comprehensiveness (PREM), independent variable Self-reported general health, multilevel linear regression

| *Random* | *intercept* |  |  |  |  |
| --- | --- | --- | --- | --- | --- |
| *Fixed effects* | Coefficient (p-value) |  |  |  |  |
| Intercept | 0.682 |  |  |  |  |
| Very good (ref) | - |  |  |  |  |
| Good | 0.0012 (<0.001) |  |  |  |  |
| Fair | 0.0028 (<0.001) |  |  |  |  |
| Poor | 0.0043 (<0.001) |  |  |  |  |
| *Random effects* |  | *Random*  Very good | *Slope*  Good | *Model*  Fair | *Variances* |
| Individual variance | 0.0006 | 0.0005 | 0.0006 | 0.0006 | 0.0006 |
| GP variance | 0.0116 | 0.0108 | 0.0116 | 0.0117 | 0.0115 |
| Country variance | 0.0093 | 0.0099 | 0.0094 | 0.0092 | 0.0080 |
| Total variance | 0.0215 |  |  |  |  |
| 0.25*variance | 0.0054 |  |  |  |  |

Table D2: Dependent variable Comprehensiveness (PREM), independent variable Having a longstanding disease, multilevel linear regression

| *Random* | *Intercept* |  |  |
| --- | --- | --- | --- |
| *Fixed effects* | Coefficient (p-value) |  |  |
| Intercept | 0.684 |  |  |
| No (ref) | - |  |  |
| Yes | 0.0033 (<0.001) |  |  |
| *Random effects* |  | *Random slope*  No | *model variances*  Yes |
| Individual variance | 0.0006 | 0.0006 | 0.006 |
| GP variance | 0.0116 | 0.0117 | 0.0115 |
| Country variance | 0.0093 | 0.0094 | 0.0092 |
| Total variance | 0.0215 |  |  |
| 0.25*variance | 0.0054 |  |  |

Table D3: Dependent variable Comprehensiveness (PREM), independent variable Patient’s age, multilevel linear regression

| *Random* | *Intercept* |  |  |  |  |
| --- | --- | --- | --- | --- | --- |
| *Fixed effects* | Coefficient (p-value) |  |  |  |  |
| Intercept | 0.682 |  |  |  |  |
| 18-40 (ref) | - |  |  |  |  |
| 41-60 | 0.0027 (p<0.001) |  |  |  |  |
| 61-75 | 0.0036 (p<0.001) |  |  |  |  |
| 76 and older | 0.0030 (p<0.001) |  |  |  |  |
| *Random effects* |  | *Random slope*  18-40 | *model variances*  41-60 | 61-75 | 76 and older |
| Individual variance | 0.0006 | 0.0006 | 0.0006 | 0.0005 | 0.0005 |
| GP variance | 0.0116 | 0.012 | 0.012 | 0.011 | 0.011 |
| Country variance | 0.0093 | 0.0099 | 0.0095 | 0.0094 | 0.0086 |
| Total variance | 0.0215 |  |  |  |  |
| 0.25*variance | 0.0054 |  |  |  |  |

Table D4: Dependent variable Comprehensiveness (PREM), independent variable Patient’s sex, multilevel linear regression

| *Random* | *Intercept* |  |  |
| --- | --- | --- | --- |
| *Fixed effects* | Coefficient (p-value) |  |  |
| Intercept | 0.684 |  |  |
| Male (ref) | - |  |  |
| Female | 0.0003 (0.192) |  |  |
| *Random effects* |  | *Random slope*  Male | *model variances*  Female |
| Individual variance | 0.0006 | 0.0006 | 0.0006 |
| GP variance | 0.0116 | 0.0117 | 0.0116 |
| Country variance | 0.0093 | 0.0094 | 0.0093 |
| Total variance | 0.0215 |  |  |
| 0.25*variance | 0.0054 |  |  |

Table D5: Dependent variable Comprehensiveness (PREM), independent variable Patient’s education, multilevel linear regression

| *Random* | *Intercept* |  |  |  |
| --- | --- | --- | --- | --- |
| *Fixed effects* | Coefficient (p-value) |  |  |  |
| Intercept | 0.685 |  |  |  |
| Low (ref) | - |  |  |  |
| Middle | -0.001 (<0.001) |  |  |  |
| High | -0.0018 (<0.001) |  |  |  |
| *Random effects* |  | *Random slope*  Low | *model variances*  Middle | High |
| Individual variance | 0.0006 | 0.0006 | 0.0006 | 0.0005 |
| GP variance | 0.0116 | 0.0115 | 0.0117 | 0.0115 |
| Country variance | 0.0093 | 0.0096 | 0.0092 | 0.0095 |
| Total variance | 0.0215 |  |  |  |
| 0.25*variance | 0.0054 |  |  |  |

Table D6: Dependent variable Comprehensiveness (PREM), independent variable Household income, multilevel linear regression

| *Random* | *Intercept* |  |  |  |
| --- | --- | --- | --- | --- |
| *Fixed effects* | Coefficient (p-value) |  |  |  |
| Intercept | 0.685 |  |  |  |
| Low (ref) | - |  |  |  |
| Middle | -0.0008 (0.001) |  |  |  |
| High | -0.0013 (0.001) |  |  |  |
| *Random effects* |  | *Random slope*  Low | *model variances*  Middle | High |
| Individual variance | 0.0006 | 0.0006 | 0.0006 | 0.0005 |
| GP variance | 0.0116 | 0.0118 | 0.0115 | 0.0101 |
| Country variance | 0.0093 | 0.0093 | 0.0093 | 0.0083 |
| Total variance | 0.0215 |  |  |  |
| 0.25*variance | 0.0054 |  |  |  |

Table D7: Dependent variable Comprehensiveness (PREM), independent variable Migrant status, multilevel linear regression

| *Random* | *Intercept* |  |  |  |
| --- | --- | --- | --- | --- |
| *Fixed effects* | Coefficient (p-value) |  |  |  |
| Intercept | 0.684 |  |  |  |
| Born in this country (ref) | - |  |  |  |
| First generation migrant | -0.0003 (0.582) |  |  |  |
| Second generation migrant | -0.0015 (0.008) |  |  |  |
| *Random effects* |  | *Random slope*  Born in this country | *model variances*  First generation migrant | Second generation migrant |
| Individual variance | 0.0006 | 0.0006 | 0.0005 | 0.0005 |
| GP variance | 0.0116 | 0.0116 | 0.0090 | 0.0093 |
| Country variance | 0.0093 | 0.0093 | 0.0079 | 0.0085 |
| Total variance | 0.0215 |  |  |  |
| 0.25*variance | 0.0054 |  |  |  |

Table D8: Dependent variable Comprehensiveness (PREM), independent variable Place of living, multilevel linear regression

| *Random* | *Intercept* |  |  |  |  |  |
| --- | --- | --- | --- | --- | --- | --- |
| *Fixed effects* | Coefficient (p-value) |  |  |  |  |  |
| Intercept | 0.678 |  |  |  |  |  |
| Big (inner) city (ref) | - |  |  |  |  |  |
| Suburbs | -0.001 (0.842) |  |  |  |  |  |
| (Small) Towns | 0.0037 (0.335) |  |  |  |  |  |
| Mixed urban-rural | 0.0091 (0.031) |  |  |  |  |  |
| Rural | 0.0183 (<0.001) |  |  |  |  |  |
| *Random effects* |  | *Random*  Big (inner) city | *Slope*  Suburbs | *Model*  (Small) towns | Variance  Mixed urban-rural | Rural |
| Individual variance | 0.0006 | 0.0006 | 0.0006 | 0.0006 | 0.0005 | 0.0005 |
| GP variance | 0.0116 | 0.013 | 0.011 | 0.012 | 0.0096 | 0.0102 |
| Country variance | 0.0091 | 0.0089 | 0.0100 | 0.0067 | 0.0058 | 0.0088 |
| Total variance | 0.0213 |  |  |  |  |  |
| 0.25*variance | 0.0053 |  |  |  |  |  |

Table E1: Dependent variable Coping after the consultation (PROM), independent variable Self-reported general health, multilevel logistic regression

| *Random* | *intercept* |  |  |  |  |
| --- | --- | --- | --- | --- | --- |
| *Fixed effects* | Coefficient (p-value) |  |  |  |  |
| Intercept | - |  |  |  |  |
| Very good (ref) | - |  |  |  |  |
| Good | 0.18 (0.002) |  |  |  |  |
| Fair | 0.075 (0.209) |  |  |  |  |
| Poor | -0.414 (<0.001) |  |  |  |  |
| *Random effects* |  | *Random*  Very good | *Slope*  Good | *Model*  Fair | *Variances*  Poor |
| Individual variance | - | - | - | - | - |
| GP variance | 0.915 | 0.65 | 0.84 | 0.82 | 0.45 |
| Country variance | 0.281 | 0.35 | 0.37 | 0.27 | 0.19 |
| Total variance^a^ | 4.49 |  |  |  |  |
| 0.25*variance | 1.12 |  |  |  |  |

^a^ Individual variance approximated by 3.29 (pi^2^/3)

Table E2: Dependent variable Coping after the consultation (PROM), independent variable Having a longstanding disease, multilevel logistic regression

| *Random* | *Intercept* |  |  |
| --- | --- | --- | --- |
| *Fixed effects* | Coefficient (p-value) |  |  |
| Intercept | - |  |  |
| No (ref) | - |  |  |
| Yes | -0.0057 (0.87) |  |  |
| *Random effects* |  | *Random slope*  No | *model variances*  Yes |
| Individual variance | - | - | - |
| GP variance | 0.915 | 0.94 | 0.81 |
| Country variance | 0.287 | 0.41 | 0.22 |
| Total variance^a^ | 4.49 |  |  |
| 0.25*variance | 1.12 |  |  |

^a^ Individual variance approximated by 3.29 (pi^2^/3)

Table E3: Dependent variable Coping after the consultation (PROM), independent variable Patient’s age, multilevel logistic regression

| *Random* | *Intercept* |  |  |  |  |
| --- | --- | --- | --- | --- | --- |
| *Fixed effects* | Coefficient (p-value) |  |  |  |  |
| Intercept | - |  |  |  |  |
| 18-40 (ref) | - |  |  |  |  |
| 41-60 | 0.217 (<0.001) |  |  |  |  |
| 61-75 | 0.419 (<0.001) |  |  |  |  |
| 76 and older | 0.434 (<0.001) |  |  |  |  |
| *Random effects* |  | *Random*  18-40 | *slope*  41-60 | *model*  61-75 | *variances*  76 and older |
| Individual variance | - | - | - | - | - |
| GP variance | 0.902 | 0.852 | 0.713 | 0.817 | 0.307 |
| Country variance | 0.284 | 0.280 | 0.338 | 0.306 | 0.240 |
| Total variance^a^ | 4.48 |  |  |  |  |
| 0.25*variance | 1.12 |  |  |  |  |

^a^ Individual variance approximated by 3.29 (pi^2^/3)

Table E4: Dependent variable Coping after the consultation (PROM), independent variable Patient’s sex, multilevel logistic regression

| *Random* | *Intercept* |  |  |
| --- | --- | --- | --- |
| *Fixed effects* | Coefficient (p-value) |  |  |
| Intercept | 0.906 |  |  |
| Male (ref) | - |  |  |
| Female | 0.146  (<0.001) |  |  |
| *Random effects* |  | *Random slope*  Male | *model variances*  Female |
| Individual variance | - | - | - |
| GP variance | 0.914 | 0.820 | 0.794 |
| Country variance | 0.287 | 0.247 | 0.316 |
| Total variance^a^ | 4.40 |  |  |
| 0.25*variance | 1.10 |  |  |

^a^ Individual variance approximated by 3.29 (pi^2^/3)

Table E5: Dependent variable Coping after the consultation (PROM), independent variable Patient’s education, multilevel logistic regression

| *Random* | *Intercept* |  |  |  |
| --- | --- | --- | --- | --- |
| *Fixed effects* | Coefficient (p-value) |  |  |  |
| Intercept | - |  |  |  |
| Low (ref) | - |  |  |  |
| Middle | -0.139 (0.003) |  |  |  |
| High | -0.158 (0.002) |  |  |  |
| *Random effects* |  | *Random slope*  Low | *model variances*  Middle | High |
| Individual variance | - | - | - | - |
| GP variance | 0.912 | 0.828 | 0.846 | 0.607 |
| Country variance | 0.283 | 0.246 | 0.297 | 0.405 |
| Total variance^a^ | 4.49 |  |  |  |
| 0.25*variance | 1.12 |  |  |  |

^a^ Individual variance approximated by 3.29 (pi^2^/3)

Table E6: Dependent variable Coping after the consultation (PROM), independent variable Household income, multilevel logistic regression

| *Random* | *Intercept* |  |  |  |
| --- | --- | --- | --- | --- |
| *Fixed effects* | Coefficient (p-value) |  |  |  |
| Intercept | - |  |  |  |
| Low (ref) | - |  |  |  |
| Middle | 0.236 (<0.001) |  |  |  |
| High | 0.047 (0.47) |  |  |  |
| *Random effects* |  | *Random slope*  Low | *model variances*  Middle | High |
| Individual variance | - | - | - | - |
| GP variance | 0.914 | 0.953 | 0.809 | 0.324 |
| Country variance | 0.291 | 0.183 | 0.377 | 0.338 |
| Total variance^a^ | 4.49 |  |  |  |
| 0.25*variance | 1.12 |  |  |  |

^a^ Individual variance approximated by 3.29 (pi^2^/3)

Table E7: Dependent variable Coping after the consultation (PROM), independent variable Migrant status, multilevel logistic regression

| *Random* | *Intercept* |  |  |  |
| --- | --- | --- | --- | --- |
| *Fixed effects* | Coefficient (p-value) |  |  |  |
| Intercept | - |  |  |  |
| Born in this country (ref) | - |  |  |  |
| First generation migrant | -0.101 (0.19) |  |  |  |
| Second generation migrant | -0.328  (<0.001) |  |  |  |
| *Random effects* |  | *Random slope*  Born in this country | *model variances*  First generation migrant | Second generation migrant |
| Individual variance |  | - | - | - |
| GP variance | 0.913 | 0.955 | 0.371 | 0.090 |
| Country variance | 0.293 | 0.287 | 0.373 | 0.195 |
| Total variance^a^ | 4.50 |  |  |  |
| 0.25*variance | 1.13 |  |  |  |

^a^ Individual variance approximated by 3.29 (pi^2^/3)

Table E8: Dependent variable Coping after the consultation (PROM), independent variable Place of living, multilevel logistic regression

| *Random* | *Intercept* |  |  |  |  |  |
| --- | --- | --- | --- | --- | --- | --- |
| *Fixed effects* | Coefficient (p-value) |  |  |  |  |  |
| Intercept | - |  |  |  |  |  |
| Big (inner) city (ref) | - |  |  |  |  |  |
| Suburbs | 0.071 0.39) |  |  |  |  |  |
| (Small) Towns | 0.093 (0.14) |  |  |  |  |  |
| Mixed urban-rural | 0.201 (0.005) |  |  |  |  |  |
| Rural | 0.403 (<0.001) |  |  |  |  |  |
| *Random effects* |  | *Random*  Big (inner) city | *Slope*  Suburbs | *Model*  (Small) towns | *Variances*  Mixed urban-rural | Rural |
| Individual variance | - | - | - | - | - | - |
| GP variance | 0.908 | 0.886 | 0.932 | 0.966 | 0.802 | 0.790 |
| Country variance | 0.284 | 0.315 | 0.318 | 0.247 | 0.269 | 0.290 |
| Total variance^a^ | 4.48 |  |  |  |  |  |
| 0.25*variance | 1.12 |  |  |  |  |  |

^a^ Individual variance approximated by 3.29 (pi^2^/3)

Table F1: Dependent variable Self-reported general health (PROM), independent variable Having a longstanding disease, multilevel linear regression

| *Random* | *Intercept* |  |  |
| --- | --- | --- | --- |
| *Fixed effects* | Coefficient (p-value) |  |  |
| Intercept | 1.97 |  |  |
| No (ref) | - |  |  |
| Yes | 0.75 (<0.001) |  |  |
| *Random effects* |  | *Random slope*  No | *model variances*  Yes |
| Individual variance | 0.482 | 0.421 | 0.512 |
| GP variance | 0.0278 | 0.034 | 0.040 |
| Country variance | 0.0367 | 0.030 | 0.058 |
| Total variance | 0.546 |  |  |
| 0.25*variance | 0.137 |  |  |

Table F2: Dependent variable Self-reported general health (PROM), independent variable Patient’s age, multilevel linear regression

| *Random* | *Intercept* |  |  |  |  |
| --- | --- | --- | --- | --- | --- |
| *Fixed effects* | Coefficient (p-value) |  |  |  |  |
| Intercept | 1.97 |  |  |  |  |
| 18-40 (ref) | - |  |  |  |  |
| 41-60 | 0.422 (<0.001) |  |  |  |  |
| 61-75 | 0.647 (<0.001) |  |  |  |  |
| 76 and older | 0.429 (<0.001) |  |  |  |  |
| *Random effects* |  | *Random*  18-40 | *slope*  41-60 | *model*  61-75 | *variances*  76 and older |
| Individual variance | 0.544 | 0.498 | 0.552 | 0.495 | 0.507 |
| GP variance | 0.032 | 0.040 | 0.043 | 0.055 | 0.058 |
| Country variance | 0.043 | 0.018 | 0.050 | 0.094 | 0.099 |
| Total variance | 0.619 |  |  |  |  |
| 0.25*variance | 0.15 |  |  |  |  |

Table F3: Dependent variable Self-reported general health (PROM), independent variable Patient’s sex, multilevel linear regression

| *Random* | *Intercept* |  |  |
| --- | --- | --- | --- |
| *Fixed effects* | Coefficient (p-value) |  |  |
| Intercept | 2.33 |  |  |
| Male (ref) | - |  |  |
| Female | 0.0265  (<0.001) |  |  |
| *Random effects* |  | *Random slope*  Male | *model variances*  Female |
| Individual variance | 0.6074 | 0.608 | 0.604 |
| GP variance | 0.0399 | 0.0429 | 0.0384 |
| Country variance | 0.0398 | 0.0289 | 0.0487 |
| Total variance | 0.6871 |  |  |
| 0.25*variance | 0.172 |  |  |

Table F4: Dependent variable Self-reported general health (PROM), independent variable Patient’s education, multilevel linear regression

| *Random* | *Intercept* |  |  |  |
| --- | --- | --- | --- | --- |
| *Fixed effects* | Coefficient (p-value) |  |  |  |
| Intercept | 2.63 |  |  |  |
| Low (ref) | - |  |  |  |
| Middle | -0.313 (<0.001) |  |  |  |
| High | -0.506 (<0.001) |  |  |  |
| *Random effects* |  | *Random slope*  Low | *model variances*  Middle | High |
| Individual variance | 0.577 | 0.566 | 0.573 | 0.551 |
| GP variance | 0.0351 | 0.0548 | 0.0445 | 0.0371 |
| Country variance | 0.0357 | 0.0545 | 0.0361 | 0.0322 |
| Total variance | 0.6482 |  |  |  |
| 0.25*variance | 0.16 |  |  |  |

Table F5: Dependent variable Self-reported general health (PROM), independent variable Household income, multilevel linear regression

| *Random* | *Intercept* |  |  |  |
| --- | --- | --- | --- | --- |
| *Fixed effects* | Coefficient (p-value) |  |  |  |
| Intercept | 2.59 |  |  |  |
| Low (ref) | - |  |  |  |
| Middle | -0.3091 (<0.001) |  |  |  |
| High | -0.5339  (<0.001) |  |  |  |
| *Random effects* |  | *Random slope*  Low | *model variances*  Middle | High |
| Individual variance | 0.5821 | 0.629 | 0.544 | 0.540 |
| GP variance | 0.0360 | 0.0633 | 0.0347 | 0.050 |
| Country variance | 0.0325 | 0.0494 | 0.0289 | 0.0195 |
| Total variance | 0.6506 |  |  |  |
| 0.25*variance | 0.16 |  |  |  |

Table F6: Dependent variable Self-reported general health (PROM), independent variable Migrant status, multilevel linear regression

| *Random* | *Intercept* |  |  |  |
| --- | --- | --- | --- | --- |
| *Fixed effects* | Coefficient (p-value) |  |  |  |
| Intercept | 2.34 |  |  |  |
| Born in this country (ref) | - |  |  |  |
| First generation migrant | 0.0335  (0.014) |  |  |  |
| Second generation migrant | -0.0179  (0.305) |  |  |  |
| *Random effects* |  | *Random slope*  Born in this country | *model variances*  First generation migrant | Second generation migrant |
| Individual variance | 0.6076 | 0.603 | 0.618 | 0.613 |
| GP variance | 0.0398 | 0.0416 | 0.0406 | 0.0323 |
| Country variance | 0.0403 | 0.0390 | 0.0789 | 0.0505 |
| Total variance | 0.6877 |  |  |  |
| 0.25*variance | 0.17 |  |  |  |

Table F7: Dependent variable Self-reported general health (PROM), independent variable Place of living, multilevel linear regression

| *Random* | *Intercept* |  |  |  |  |  |
| --- | --- | --- | --- | --- | --- | --- |
| *Fixed effects* | Coefficient (p-value) |  |  |  |  |  |
| Intercept | 2.33 |  |  |  |  |  |
| Big (inner) city (ref) | - |  |  |  |  |  |
| Suburbs | -0.0129 (0.388) |  |  |  |  |  |
| (Small) Towns | 0.0149 (0.210) |  |  |  |  |  |
| Mixed urban-rural | 0.0352 (0.008) |  |  |  |  |  |
| Rural | 0.0405 (0.002) |  |  |  |  |  |
| *Random effects* |  | *Random*  Big (inner) city | *Slope*  Suburbs | *Model*  (Small) towns | *Variances*  Mixed urban-rural | Rural |
| Individual variance | 0.6076 | 0.610 | 0.584 | 0.620 | 0.611 | 0.602 |
| GP variance | 0.0396 | 0.042 | 0.0278 | 0.0386 | 0.0253 | 0.0442 |
| Country variance | 0.0396 | 0.0327 | 0.0429 | 0.0448 | 0.0451 | 0.0566 |
| Total variance | 0.6868 |  |  |  |  |  |
| 0.25*variance | 0.17 |  |  |  |  |  |

Table G1: Variances at country and GP level (% of total variance) for potential case-mix adjusters, multilevel multinomial or logistic regression; first category as reference category. All variances are significantly different from zero.

| Potential case-mix adjuster | % of total variance at country | % of total variance at GP |
| --- | --- | --- |
| Self-reported general health ^a^   - Good | 3.26 | 10.58 |
| - Fair | 8.96 | 13.16 |
| - Poor | 13.91 | 16.30 |
| Having a longstanding disease ^b^   - Yes | 3.85 | 5.11 |
| Patient’s age ^a^   - 41-60 | 1.61 | 8.95 |
| - 60-75 | 5.87 | 15.85 |
| - 76 and older | 10.34 | 20.75 |
| Patient’s sex ^b^   - female | 0.88 | 2.63 |
| Patient’s education ^a^   - middle | 8.73 | 10.14 |
| - high | 17.05 | 14.08 |
| Household income ^a^   - middle | 7.48 | 8.97 |
| - high | 13.22 | 15.48 |
| Migrant status ^b, c^   - first and second generation migrant | 27.56 | 17.90 |
| Place of living ^a^   - suburbs | 17.71 | * |
| - (small) towns | 7.76 | * |
| - mixed urban-rural | 12.88 | * |
| - rural | 17.31 | * |

^a^ Multinomial regression

^b^ Logistic regression

^c^ First and second generation were combined because of small numbers

* Not calculated at patient level, because place of living was approximated by location of the practice
